# Charting the twist-to-bend ratio of plant axes

**DOI:** 10.1098/rsif.2022.0131

**Published:** 2022-06-22

**Authors:** Steve Wolff-Vorbeck, Olga Speck, Max Langer, Thomas Speck, Patrick W. Dondl

**Affiliations:** ^1^ Department for Applied Mathematics, University of Freiburg, 79104 Freiburg, Germany; ^2^ Plant Biomechanics Group @ Botanic Garden, Faculty of Biology, University of Freiburg, 79104 Freiburg, Germany; ^3^ Cluster of Excellence *liv*MatS @ FIT – Freiburg Center for Interactive Materials and Bioinspired Technologies, University of Freiburg, 79104 Freiburg, Germany

**Keywords:** bending modulus, finite-element method, flexural rigidity, torsional modulus, torsional rigidity, twist-to-bend ratio

## Abstract

During the evolution of land plants many body plans have been developed. Differences in the cross-sectional geometry and tissue pattern of plant axes influence their flexural rigidity, torsional rigidity and the ratio of both of these rigidities, the so-called twist-to-bend ratio. For comparison, we have designed artificial cross-sections with various cross-sectional geometries and patterns of vascular bundles, collenchyma or sclerenchyma strands, but fixed percentages for these tissues. Our mathematical model allows the calculation of the twist-to-bend ratio by taking both cross-sectional geometry and tissue pattern into account. Each artificial cross-section was placed into a rigidity chart to provide information about its twist-to-bend ratio. In these charts, artificial cross-sections with the same geometry did not form clusters, whereas those with similar tissue patterns formed clusters characterized by vascular bundles, collenchyma or sclerenchyma arranged as one central strand, as a peripheral closed ring or as distributed individual strands. Generally, flexural rigidity increased the more the bundles or fibre strands were placed at the periphery. Torsional rigidity decreased the more the bundles or strands were separated and the less that they were arranged along a peripheral ring. The calculated twist-to-bend ratios ranged between 0.85 (ellipse with central vascular bundles) and 196 (triangle with individual peripheral sclerenchyma strands).

## 1. Introduction

### 1.1. Body plans of plants

**Figure 1.**
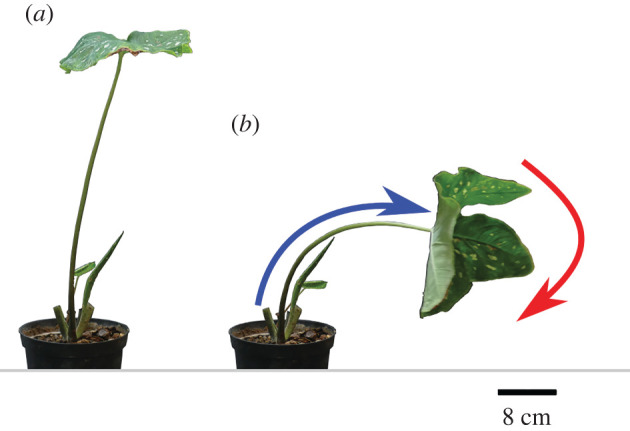
Peltate leaf of *Caladium bicolor*. (*a*) The petiole stands upright under windless conditions. (*b*) The petiole bends (blue arrow) and twists (red arrow) simultaneously under wind load.

In their natural environment, plants are exposed to a wide range of bending and torsional loads. Figure 1*a* shows an upright foliage leaf in a windless situation with a slender and tapered petiole that is rigid enough to support the huge lamina against gravitational force and even a multiple of its own weight [1]. Figure 1*b* depicts the same leaf under wind loads, with the petiole being flexible enough to bend and twist simultaneously, resulting in a streamlined shape that ultimately prevents the leaf from damage. This mechanical behaviour of the petiole is influenced by both its flexural rigidity and its torsional rigidity, the trade-off of which can be elegantly expressed by the dimensionless twist-to-bend ratio [2].

Generally, the biomechanical performance of plants is determined by their so-called general body plan, which is a set of morphological features common to many members of a phyllum [3]. The body plan of petioles includes the cross-sectional geometry, the tapering mode and the three-dimensional distribution of various tissues. These aspects form the basis for a straightforward description of plant biomechanics.

### 1.2. Structural properties of plant axes

The flexural rigidity (***Ē****I* (Nmm^2^)) and torsional rigidity ($\bar{G}$*K* (Nmm^2^)) of a plant axis are structural properties composed of its geometric properties (axial second moment of area *I* (m^4^), torsion constant = torsional second moment of area *K* (m^4^)) and its effective material properties (effective bending elastic modulus ***Ē*** (Nm^−2^), effective torsional modulus $\bar{G}$ (Nm^−2^)). In this context, the term ‘effective’ describes the material properties of the entire axis, which are a function of the material properties of the individual tissues involved, their respective volume fraction and their three-dimensional arrangement. In the past, the term ‘structural’ was sometimes used instead of ‘effective’ [4]. Most petioles of foliage leaves are easier to twist than to bend, resulting in high twist-to-bend ratios (***Ē****I*/$\bar{G}$*K* (−)) [2]. In rare cases, such as the transition zone between the petiole and the lamina of foliage leaves, plant axes exhibit twist-to-bend ratios of less than 1.0; this means that they are easier to bend than to twist [5].

### 1.3. Geometric properties of plant axes

The cross-sectional geometry of a plant stem, such as a circle, ellipse, triangle, square or U-profile, influences its biomechanical performance. For bending loads, the axial second moment of area *I* is the relevant geometric property. For torsional loads, the polar second moment of area (*J* (m^4^)) or the torsion constant (*K* (m^4^)) are of geometrical relevance. Since the polar second moment of area is calculated as the sum of the axial second moment of area in the *x*-direction and *y*-direction (*J* = *I*_*x*_ + *I*_*y*_), the ratio of *I*/*J* (–) can never exceed 1.0. For circular cross-sections *J* and *K* are identical. For all other geometries, the torsion constant *K* is smaller than *J*, and is sometimes even a small fraction of the polar second moment of area [6,7]. Consequently, *I*/*K* can become larger than 1.0 and thus can contribute to high twist-to-bend ratios. The U-profiled petioles of *Hosta* x *tardiana* ‘El Niño’ (hereafter *H. tardiana*) exhibit median values of *I*/*K* = 1.08, which contribute to median twist-to-bend ratios of $\bar{E}I/\bar{G}K=23.66$ [1] (see also the brief description in electronic supplementary material, S1 and table S1.1).

It is well known that most plant axes exhibit a taper in the apical direction, which means that their diameter changes along the length of the axis. The twist-to-bend ratio, however, is a dimensionless variable that is invariant under scaling of the diameter of the cross-section, since both the torsional and the flexural rigidity obtain the same scale factor. Since—for this study—we are mainly concerned with the twist-to-bend ratio, we thus exclusively consider the cross-sectional geometry of a plant stem, assuming that the stem is a simple beam with constant geometry and tissue pattern. We note that our model remains valid if the change in cross-sectional geometry is on a length scale much larger than the diameter, in which case one simply obtains a slowly varying twist-to-bend ratio along the plant axis, as seen, for example, along the petiole of *Musa* sp. [8].

### 1.4. Mechanical properties of plant axes

The biomechanical performance of each plant stem also depends on the tissues that compose it. From a mechanical viewpoint, each plant axis is characterized by both anatomical heterogeneity through a specific three-dimensional arrangement of various tissues and mechanical anisotropy through the various mechanical properties of the individual tissues. Plant tissues include dermal tissues (e.g. epidermis, periderm, hypodermis), ground tissues (e.g. parenchyma, chlorenchyma, hydrenchyma), vascular tissues (e.g. vascular bundles including xylem and phloem) and strengthening tissues (e.g. collenchyma and sclerenchyma), which differ significantly in their mechanical properties.

Table 1 shows that the values for the elastic modulus *E* ((Nmm^2^) = (MPa)) of individual plant tissues differ by orders of magnitude. The same holds true for their shear modulus *G*, which (in the framework of isotropic linearized elasticity) is related to the elastic modulus by Poisson’s ratio ν (–), the latter being the transverse elongation divided by the amount of axial compression [7]. For most individual plant materials, the ratio of *E* to *G* lies between 2 and 3 if Poisson’s ratio is between 0 and 0.5. However, for entire plant structures ***Ē*** ≫ $\bar{G}$, the ratio of the effective bending elastic modulus and effective torsional modulus (***Ē***/$\bar{G}$ (–)) of the plant axes is significantly higher and thus contributes to high twist-to-bend ratios. For example, the almost circular petioles of *Caladium bicolor* exhibit a median ratio of ***Ē***/$\bar{G}$ = 63.73, which markedly contributes to the median twist-to-bend ratio of ***Ē****I*/$\bar{G}$*K* = 39.19 [1] (see also the brief description in electronic supplementary material, S1 and table S1.1).

**Table 1.** Elastic bending modulus *E* of individual plant tissues derived from the literature [9]. *Wood can differ significantly in the longitudinal, tangential and radial directions.

tissue	*E* (MPa)	references
sclerenchyma	24 500 − 45 000	[7,10,11]
wood (sec. xylem)*	2600–16 000	[10]
collenchyma	1000–2600	[7,10,12]
vascular bundles	30–840	[7,10,13]
epidermis + periderm	350–500	[14]
epidermis	3–250	[10,13,15]
parenchyma (non-lignified)	5–100	[10]

### 1.5. Twist-to-bend ratios of plant axes

In 1992, Vogel presented the relationship between the cross-sectional geometry and twist-to-bend ratio of petioles and plant stems [2]. He measured the flexural and torsional rigidity of circular and non-circular herbaceous stems and petioles. Specimens with non-circular cross-sections had higher twist-to-bend ratios and thus were relatively more flexible in twisting than in bending. However, all twist-to-bend ratios were in the single-digit range. In 1993, Ennos investigated the mechanics of the triangular flower stalks of *Carex acutiformis* and reported twist-to-bend ratios in the two-digit range between 22 and 51 [16]. In 2003, Etnier mapped ideal beams and biological axes into a size-normalized mechanospace defined by flexural and torsional rigidity. Based on assumed material values and chosen cross-sectional geometries (circle, ellipse), she defined boundaries that mirrored possible extremes for biological beams and found that 53 of the 57 selected specimens (=93%) fell within the bounded region [17]. In addition to circular and elliptic geometries, petioles exhibit lengthwise grooving with high twist-to-bend ratios of up to 100 [1,18]. The twist-to-bend ratios of U-profiled petioles of banana leaves (*Musa* sp.) range between 40 and 100 [19,20]. A numerical sensitivity analysis [8] has shown that plant axes tend to form grooves and U-profiles when high twist-to-bend ratios are mechanically favourable.

The triangular flower stalks of *Carex pendula* (hereafter *C. pendula*) have twist-to-bend ratios of up to 400, which are the highest values ever measured in stems of herbaceous plants [21] (see also the brief description in electronic supplementary material, S1 and table S1.1).

### 1.6. Tissue patterns of plant axes

With respect to the three-dimensional arrangement of plant tissues, we refer to a review focusing on the form–function relationship of large foliage leaves from a mechanical perspective [22]. Niklas related stress distribution to tissue distribution in the circular plant axis. The tensile and compressive stress components that result from bending and torsional shear stress reach their maximum intensities at the surface of the petiole. In contrast, the shear stresses resulting from bending reach their maximum intensities at the centre of a circular cross-section [7,22]. Dependent on the general body plan of the respective plants, these stresses are accommodated either by living and hydrostatic (= turgor dependent) tissues such as parenchyma and collenchyma or by dead and rigid tissues such as the xylem of the vascular bundles (tracheids, vessels) and sclerenchyma [22].

**Figure 2.**
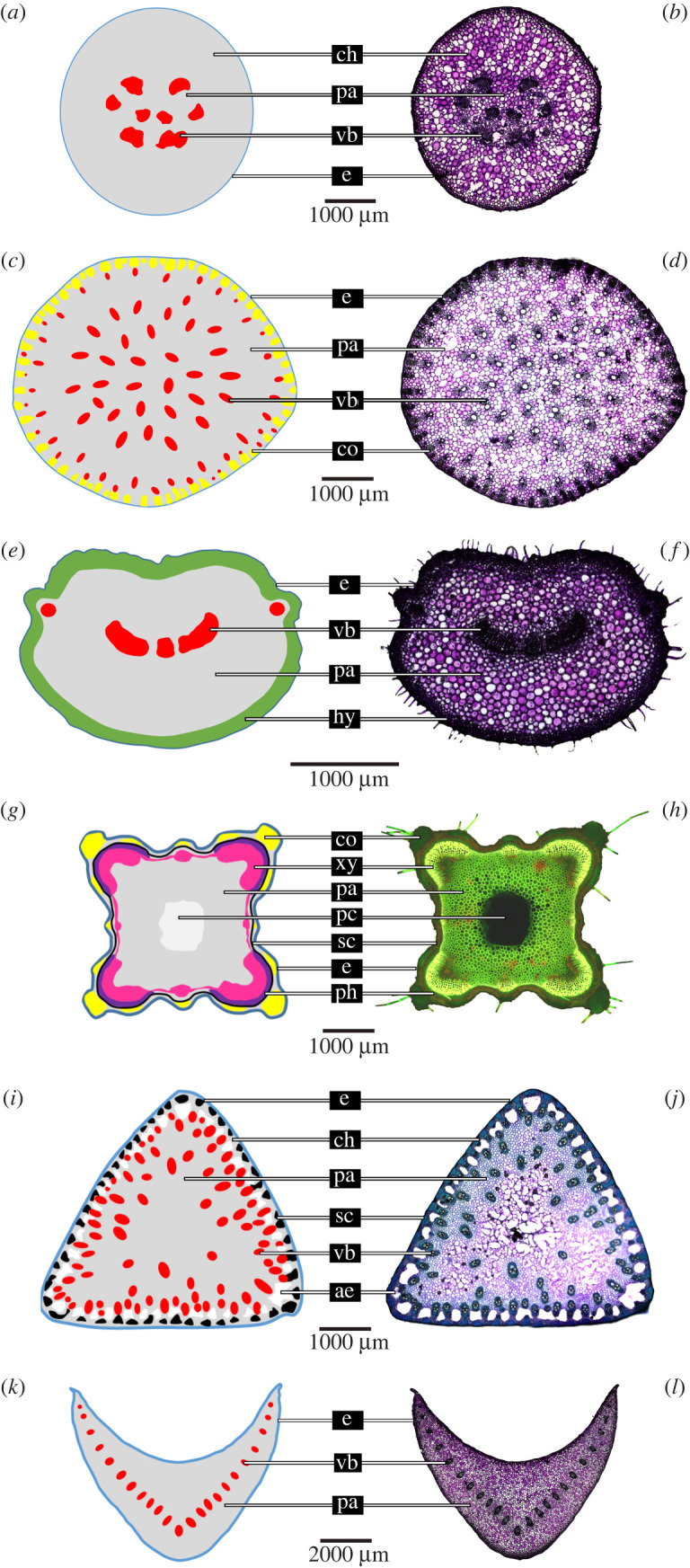
Cross-sections of plant axes. Petioles of (*a*,*b*) *Pilea peperomioides*, (*c*,*d*) *Caladium bicolor* and (*e*,*f* ) *Hemigraphis alternata*, flower stalks of (*g*,*h*) *Leonurus cardiaca* and (*i*,*j*) *Carex pendula* and petioles of (*k*,*l*) *Hosta* x *tardiana* ‘El Niño’. Left side (*a*,*c*,*e*,*g*,*i*,*k*): schematic drawings; abbreviations and colour code: ae, aerenchyma (white); co, collenchyma fibres (yellow); ch, chlorenchyma (grey); e, epidermis (blue); hy, collenchymatous hypodermis (green); pa, parenchyma (grey); pc, pith cavity (light grey); ph, phloem (purple); sc, sclerenchyma fibres (black); vb, vascular bundles (red); xy, secondary xylem (magenta). Right side: (*b*,*d*,*f*,*j*,*l*) thin sections stained with toluidine blue O highlighting lignified cell walls in blue and non-lignified cell walls in red; (*h*) thin section stained with acridine orange highlighting lignified tissues in bright yellow-green.

Figure 2 illustrates various transverse sections of plant axes with differing cross-sectional geometries and tissue patterns. Based on stained thin sections from previous studies [1,21,23–25], corresponding schematic drawings were created depicting the cross-sectional geometry of the plant axes and the distribution of the tissues involved. These plant examples were selected because their geometric, mechanical and structural properties were available for discussion of the results of the simulations of this study (electronic supplementary material, S1 and table S1.1). Moreover, a brief description of each plant species is given in electronic supplementary material, S1.

### 1.7. Aim of the study

To date, no mathematical models have been developed that simultaneously account for the influence of cross-sectional geometry and tissue pattern on the flexural rigidity, torsional rigidity and twist-to-bend ratio of plant axes. However, quantification of the influence of individual properties and their combinations can provide general indications concerning the functional morphology of plants. Furthermore, rigidity charts might form the basis for the development of bioinspired designs by engineers.

Our aim has been to find answers to the following scientific question: How do the cross-sectional geometry and/or the arrangement of vascular bundles or strengthening tissues influence the flexural rigidity, the torsional rigidity and thus the twist-to-bend ratio of plant axes? We have addressed four main aspects: (i) the design of artificial cross-sections differing in geometry and/or the tissue pattern of vascular bundles, collenchyma fibre strands and sclerenchyma fibre strands; (ii) the development of a mathematical model for numerical determination of flexural rigidity and torsional rigidity of the artificial cross-sections; (iii) the arrangement of the artificial cross-sections in rigidity charts; and (iv) an analysis of flexural and torsional rigidity as a function of the number of peripheral individual strands.

## 2. Material and methods

### 2.1. Mathematical modelling

As in previous work [8,9], we use methods from linearized elasticity and Saint-Venant's theory of pure torsion of non-homogeneous elastic beams. We therefore describe a plant stem as a long thin elastic rod with domain B=Ω×(0,L) of length *L* and simply connected cross-section Ω remaining constant along the longitudinal axis. The heterogeneity of the cross-section Ω is described by spatially dependent mechanical moduli that are determined by the elastic modulus *E*(*x*, *y*) and the shear modulus *G*(*x*, *y*) of the specific material of the tissue located at position (x,y)∈Ω. Unless the cross-section Ω consists of one single material, the quantities *E* and *G* thus differ from the effective bending elastic modulus ***Ē*** and the effective torsional modulus $\bar{G}$.

Assuming now that *E*(*x*, *y*) and *G*(*x*, *y*) are piece-wise constant in Ω and following rigorous results from [26], the minimal and maximal flexural rigidity along the principal axes are given by

$$D_{\max/\min}=\left(D_{\mathrm{mean}}\pm \sqrt{\frac{{\left(D_{x}-D_{y}\right)}^{2}}{4}+D_{xy}^{2}}\right),$$

where

$$\begin{array}{rl} & D_{x}=\int_{\Omega }E(x,y){\hat{x}}^{2}\;dxdy,\;D_{y}=\int_{\Omega }E(x,y){\hat{y}}^{2}\;dxdy,\\ & D_{xy}=\int_{\Omega }E(x,y)\hat{x}\hat{y}\;dxdy, \end{array}$$

with centroids

$$\hat{y}=y-\frac{\int_{\Omega }E(x,y)y\;dxdy}{\int_{\Omega }\;E(x,y)\;dxdy},\;\hat{x}=x-\frac{\int_{\Omega }E(x,y)x\;dxdy}{\int_{\Omega }E(x,y)\;dxdy},$$

and spatially dependent elastic modulus *E*(*x*, *y*).

Torsional rigidity, on the other hand, can be expressed by Prandtl’s stress function *ϕ*(*x*, *y*) (e.g. [27,28]), satisfying

2.1 $$\begin{array}{cc} & \nabla \cdot \left(\frac{1}{G(x,y)}\nabla \phi \right)=-2,\text{in}\;\Omega ,\\ & \phi =0\;\text{on}\;\partial \Omega , \end{array}$$

with the spatially dependent shear modulus *G*(*x*, *y*). Using the stress function *ϕ*, the torsional rigidity is therefore given by

$$D_{z}=2\int_{\Omega }\phi \;dxdy.$$

From equation (2.1), it follows that, for homogeneous elastic beams, the quantities *D*_*z*_ and *D*_max/min_ depend linearly on the constants *E* and *G* and thus coincide with the quantities ***Ē****I* and $\;\bar{\;G}$*K* described above.

Usually, of course, the minimal flexural rigidity *D*_min_ is the decisive factor for the mechanical suitability of a given design. In the following, we are therefore concerned with the evaluation and comparison of the twist-to-bend ratio *D*_min_/*D*_*z*_ of various cross-sections with different types and shapes of reinforcing materials.

In order to compute the rigidities numerically, we normalize the elastic moduli (*E*_est_) of all contained materials with respect to the elastic modulus of sclerenchyma, i.e. we set *E*_norm_ = 1 for sclerenchyma and obtain the normalized elastic moduli of the other materials by appropriate scaling; see table 1. Further, we assume a constant Poisson’s ratio ν for all materials involved and compute the normalized torsional modulus *G*_norm_ as

$$G_{\mathrm{norm}}=\frac{E_{\mathrm{norm}}}{2(1+\nu )},$$

for a given elastic modulus *E*_norm_. Assumption of a constant Poisson’s ratio is reasonable because the value range ν ∈ [0.2, 0.5] is typical for many plant axes [29] and, thus, a change in ν among the materials is negligible for our model. Hereafter, we set ν = 0.35, which is an appropriate choice for herbaceous plants [13,29].

Once Prandtl’s stress function *ϕ* is determined from equation (2.1), numerical integration suffices for the computation of the rigidities *D*_*z*_ and *D*_min_. Therefore, we have employed P1 triangular finite-element discretizations of five reference cross-sections including various types of materials and have thereby solved the elliptic equation (2.1) numerically. The various materials were described by functions *E*(*x*, *y*) and *G*(*x*, *y*) of the elastic and shear modulus of the certain materials determined on the mesh points of the discretization. The implementation of this standard finite-element method (C + +-code) is made available in electronic supplementary material, S2. Raw data of the mathematical model are given in electronic supplementary material, S3 and table S3.1.

One should note that, owing to the high contrast of material parameters in some plants studied here, we reach the bounds of the regions of validity for our torsion and bending models. However, as our main objective is a grouping of reinforcement strategies in terms of system parameters, more general trends for effective properties suffice. In particular, the ‘winning’ strategies in terms of the twist-to-bend ratio (i.e. *C. pendula*) are reliably predicted. A more detailed quantitative comparison is beyond the scope of this work, as then other material properties such as turgor, anisotropy or viscoelasticity would also have to be considered.

### 2.2. Artificial cross-sections

**Figure 3.**
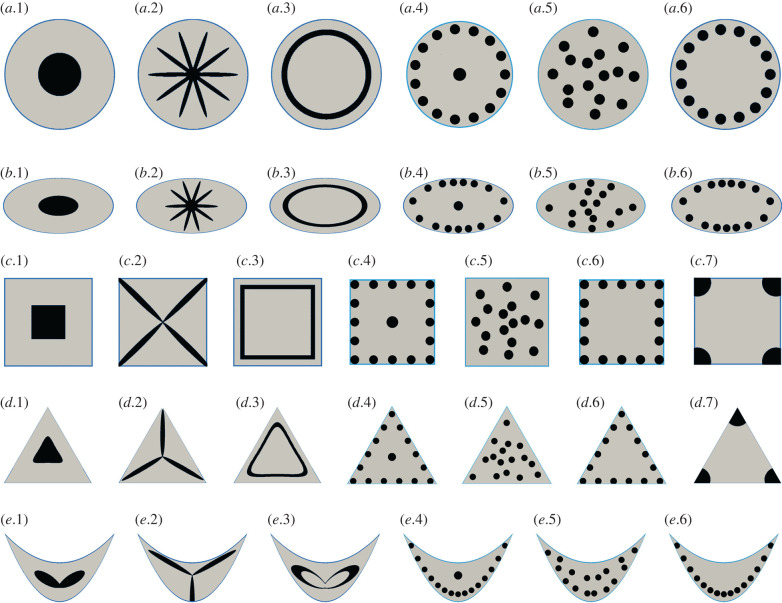
Artificial cross-sections. Each cross-section consists of an outer epidermis (blue), parenchymatous ground tissue (light grey) and vascular bundles or collenchyma or sclerenchyma (black). In diverse geometries, (*a*) circle, (*b*) ellipse, (*c*) square, (*d*) triangle, (*e*) U-profile, a variety of tissue patterns created by the vascular bundles or strengthening tissues are embedded in the parenchyma: *x*.1 central position, *x*.2 star-shaped, *x*.3 closed peripheral ring, *x*.4 central position and individual strands in the periphery, *x*.5 randomly scattered, *x*.6 individual strands in the periphery, *x*.7 corner position.

The differences in the geometry of the entire axis, the multitude of possible plant tissues involved together with their individual mechanical properties and the great variety of possible tissue arrangements within the plant axes result in ever new combinations and are thus the basis for the plethora of body plans of plants. For systematic analyses, we have designed artificial cross-sections differing in their *cross-sectional geometry* (e.g. circle, ellipse, triangle, square, U-profile) and/or *tissue pattern* by means of the arrangement of vascular bundles, collenchyma or sclerenchyma (e.g. central, star-shaped, ring, scattered, corner) (figure 3).

Our mathematical model simultaneously provides information concerning the influence of the cross-sectional geometry and tissue pattern on flexural rigidity and torsional rigidity and on the twist-to-bend ratio (see also electronic supplementary material, S3 and table S3.1). This allows us to place each artificial cross-section in a rigidity landscape, which is defined by the flexural and torsional rigidity. The respective place in the rigidity chart provides information about the trade-off between rigidity in bending and torsion and, thus, about the twist-to-bend ratio.

1. *Artificial cross-section* is an umbrella term for the following three types of designed cross-sections.
  1.1. *Standard cross-sections* were designed by means of *vascular tissue*, parenchyma and epidermis, three tissues that are necessary for a vascular plant. Each tissue has a characteristic elastic modulus *E* (table 1) and a fixed percentage of cross-sectional area *A*
    — epidermis: *E* = 50 (MPa), A=1%
    — parenchyma: *E* = 20 (MPa), A=84%
    — *vascular bundles*: *E* = 1 (GPa), A=15%
  1.2. *Collenchyma-modified cross-sections* were created by completely replacing vascular bundles with strands of collenchyma fibres:
    — epidermis: *E* = 50 (MPa), A=1%
    — parenchyma: *E* = 20 (MPa), A=84%
    — *collenchyma*: *E* = 2.5 (GPa), A=15%
  1.3. *Sclerenchyma-modified cross-sections* were created by completely replacing vascular bundles with strands of sclerenchyma fibres:
    — epidermis: *E* = 50 (MPa), A=1%
    — parenchyma: *E* = 20 (MPa), A=84%
    — *sclerenchyma*: *E* = 45 (GPa), A=15%
2. In order to assess the *sensitivity of the twist-to-bend ratio to the number of fibres*, we simulated design *x*.6 with increasing numbers of individual vascular bundles or strands of collenchyma or sclerenchyma. Similarly, each tissue has the above-mentioned characteristic elastic modulus *E* (table 1) and fixed percentage of cross-sectional area *A*. Circles, ellipses, triangles and U-profiles start with three bundles/strands, whereas squares start with four bundles/strands. The number of individual bundles/strands was increased step by step: circles and ellipses one per step, U-profiles two per step, triangles three per step and squares four per step. The maximum number was reached when the bundles/strands were still present individually and had not yet merged.

## 3. Results

### 3.1. Twist-to-bend ratios of artificial cross-sections

Table 2 presents the calculated twist-to-bend ratios of the artificial cross-sections with vascular bundles, collenchyma strands and sclerenchyma strands (figure 3). We also calculated the change factors of the twist-to-bend ratios of the fibre-modified cross-sections in relation to the standard cross-sections with vascular bundles. The change factors of the collenchyma-modified cross-sections ranged between 0.95 and 2.23. The change factors of the sclerenchyma-modified cross-sections were between 0.91 and 14.64. Different change factors were found depending on the design, indicating that the designs do not scale linearly with the change of elastic modulus. The twist-to-bend ratios of designs *x*.1 (central position) and *x*.3 (peripheral closed ring) are similar even when collenchyma or sclerenchyma is used instead of vascular bundles (change factor: ≈1.0). By contrast, design *x*.6 (individual strands in the periphery) for the circle, ellipse and U-profile consistently show the highest values of twist-to-bend ratio for the standard cross-sections and fibre-modified cross-sections. This is different from the square and triangular cross-sections, where a design change takes place with regard to the highest twist-to-bend ratios. Design *x*.7 (corner position) has the highest twist-to-bend ratios for standard and collenchyma-modified cross-sections, whereas design *x*.6 (individual strands in the periphery) shows the highest twist-to-bend ratios for the sclerenchyma-modified cross-sections. For design *x*.6, the influence of the number of single strands on the twist-to-bend ratio plays a major role (see §3.3).

**Table 2.** Twist-to-bend ratios calculated for the artificial cross-sections shown in figure 3. Vascular bundles of the standard cross-sections were replaced either by strands of collenchyma fibres or by sclerenchyma fibres. The highest value for each geometry is shown in italics. Twist-to-bend ratios of the fibre-modified cross-sections were normalized to the value of the standard cross-sections. Resulting *change factors* indicate an increase (greater than 1.0) or decrease (less than 1.0) of the twist-to-bend ratio.

		vasc. bundles	collenchyma	sclerenchyma	change factor	change factor
geometry	artificial design	twist-to-bend ratio *D*_min_/*D*_*z*_	twist-to-bend ratio *D*_min_/*D*_*z*_	twist-to-bend ratio *D*_min_/*D*_*z*_	collenchyma/vasc. bundles	sclerenchyma/vasc. bundles
circle	(*a*.1)	1.36	1.37	1.37	1.00	1.01
	(*a*.2)	4.53	9.18	66.36	2.03	14.64
	(*a*.3)	1.37	1.39	1.38	1.01	1.01
	(*a*.4)	8.56	19.09	124.23	2.23	14.51
	(*a*.5)	5.91	12.67	73.05	2.14	12.36
	(*a*.6)	*9.12*	*20.13*	*131.62*	2.21	14.43
ellipse	(*b*.1)	0.85	0.86	0.86	1.00	1.00
	(*b*.2)	2.99	5.95	30.19	1.99	10.10
	(*b*.3)	0.86	0.86	0.87	1.00	1.00
	(*b*.4)	6.57	14.52	88.11	2.21	13.50
	(*b*.5)	4.90	10.40	53.92	2.12	11.00
	(*b*.6)	*6.84*	*15.24*	*92.29*	2.23	13.50
square	(*c*.1)	1.54	1.50	1.45	0.98	0.95
	(*c*.2)	10.76	23.38	147.72	2.17	13.73
	(*c*.3)	1.77	1.79	1.80	1.01	1.02
	(*c*.4)	11.07	24.49	154.40	2.21	13.95
	(*c*.5)	6.52	13.46	71.89	2.06	11.02
	(*c*.6)	11.68	25.98	*165.11*	2.22	14.14
	(*c*.7)	*15.45*	*29.93*	80.07	1.94	5.18
triangle	(*d*.1)	2.15	2.11	2.05	0.98	0.95
	(*d*.2)	16.49	34.42	143.70	2.09	8.71
	(*d*.3)	2.18	2.18	2.17	1.00	1.00
	(*d*.4)	15.72	34.42	181.23	2.17	11.53
	(*d*.5)	9.19	18.62	87.86	2.03	9.56
	(*d*.6)	16.52	36.08	*195.90*	2.18	11.86
	(*d*.7)	*26.31*	*45.79*	90.02	1.74	3.42
U-profile	(*e*.1)	2.41	2.29	2.19	0.95	0.91
	(*e*.2)	9.72	19.05	70.76	1.96	7.28
	(*e*.3)	1.95	2.10	2.44	1.07	1.25
	(*e*.4)	12.81	26.35	113.86	2.06	8.89
	(*e*.5)	11.55	23.20	90.71	2.01	7.85
	(*e*.6)	*13.60*	*28.07*	*125.67*	2.06	9.24

### 3.2. Rigidity landscapes of artificial cross-sections

**Figure 4.**
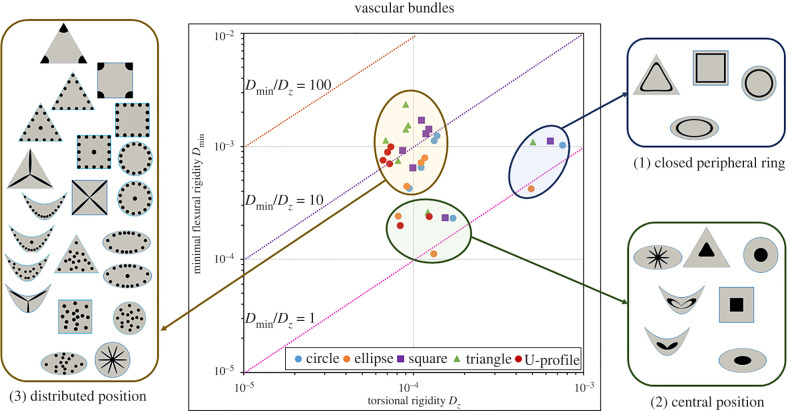
Rigidity chart of standard cross-sections with various patterns of *vascular bundles*. Minimal flexural rigidity (*D*_min_) is given as a function of torsional rigidity (*D*_*z*_) on a logarithmic scale. Each artificial cross-section is placed in the chart according to its twist-to-bend ratio. Dashed lines equal twist-to-bend ratios of 1 (magenta), 10 (violet) or 100 (brown). Three clusters were defined in terms of the arrangement of the vascular bundles: (1) closed peripheral ring, (2) central position and (3) distributed position (= individual bundles scattered throughout the parenchyma or in the periphery and slender bundles). In each of the three clusters, the angular geometries with two, three or four corners exhibit the highest twist-to-bend ratios.

**Figure 5.**
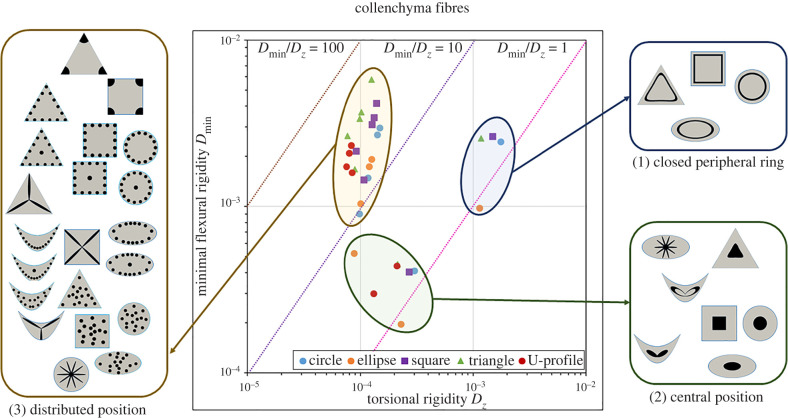
Rigidity chart of collenchyma-modified cross-sections with various patterns of *collenchyma strands*. Minimal flexural rigidity (*D*_min_) is given as a function of torsional rigidity (*D*_*z*_) on a logarithmic scale. Each artificial cross-section is placed in the chart according to its twist-to-bend ratio. Dashed lines equal twist-to-bend ratios of 1 (magenta), 10 (violet) or 100 (brown). Three clusters were defined in terms of the arrangement of the collenchyma strands: (1) closed peripheral ring, (2) central position and (3) distributed position (= individual bundles scattered throughout the parenchyma or in the periphery and slender bundles). The highest twist-to-bend ratios exhibit geometries with corners and scattered strands. In each of the three clusters, the angular geometries with two, three or four corners exhibit the highest twist-to-bend ratios.

**Figure 6.**
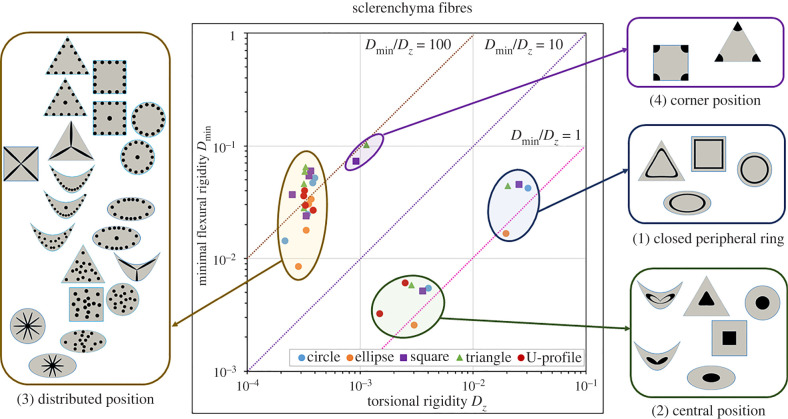
Rigidity chart of sclerenchyma-modified cross-sections with various patterns of *sclerenchyma strands*. Minimal flexural rigidity (*D*_min_) is given as a function of torsional rigidity (*D*_*z*_) on a logarithmic scale. Each artificial cross-section is placed in the chart according to its twist-to-bend ratio. Dashed lines equal twist-to-bend ratios of 1 (magenta), 10 (violet) or 100 (brown). Each artificial cross-section is placed in the chart according to its twist-to-bend ratio. Four clusters were defined in terms of the arrangement of the sclerenchyma strands: (1) closed peripheral ring, (2) central position, (3) distributed position (= individual bundles scattered throughout the parenchyma or in the periphery and slender bundles), (4) corner position.

Each artificial design (figure 3) of the standard cross-sections (figure 4), collenchyma-modified cross-sections (figure 5) and sclerenchyma-modified cross-sections (figure 6) was placed into a rigidity chart defined by the minimal flexural rigidity *D*_min_ as a function of the torsional rigidity *D*_*z*_. The position in the chart corresponds to the respective twist-to-bend ratio. Furthermore, a rigidity chart containing all cross-sections is presented in electronic supplementary material, S4 and figure S4.1.

Similarities and dissimilarities found in the three rigidity charts can be summarized as follows.

— Cross-sections with the same geometry, such as the circle, ellipse, square, triangle or U-profile, did not cluster, i.e. artificial cross-sections with different geometries were found scattered on the rigidity charts.
— Independent of whether vascular bundles, collenchyma or sclerenchyma strands were used for the modelling, two designs had a twist-to-bend ratio of less than 1.0. The ellipse with a closed ring of bundles/strands in the periphery (*b*.1) and the ellipse with a central position of bundles/strands (*b*.3) were stiffer in torsion than in bending (over the small side of the ellipse).
— Two clusters clearly emerge in all charts and are characterized by the following tissue pattern: cross-sections with a central position of bundles/strands (*x*.1) and cross-sections with a closed peripheral ring of bundles/strands (*x*.3). Although the designs of the two clusters differ in both flexural and torsional rigidity, they have comparable low values of the twist-to-bend ratio. Ultimately, this is the consequence of moving the positions of the artificial designs along the diagonal lines in the rigidity chart, which represent fixed values of the twist-to-bend ratio.
— All remaining designs differ considerably in the values of the minimum flexural rigidity, whereby the values of the torsional rigidity among each other are similar. Since angular designs (triangles, squares and U-profiles) and circular cross-sections with individual strands in the periphery (*x*.4 and *x*.6) have high values of the minimal flexural rigidity, they exhibit high twist-to-bend ratios.
— The torsional rigidity of the cluster characterized by a closed peripheral ring of bundles/strands (*x*.3) is markedly higher than the torsional rigidity of all other designs.
— The torsional rigidity of the clusters having designs with a central position (*x*.1) and designs with individual bundles/strands is similar in terms of vascular bundles and collenchyma fibres. By contrast, with respect to sclerenchyma fibres, the torsional rigidity of the cluster with individual strands is markedly smaller than that of the cluster with central position designs.
— The square and triangular designs with the sclerenchyma positioned in the corners (*x*.7) form a fourth cluster.

### 3.3. Twist-to-bend ratio as a function of the bundle/strand number

**Figure 7.**
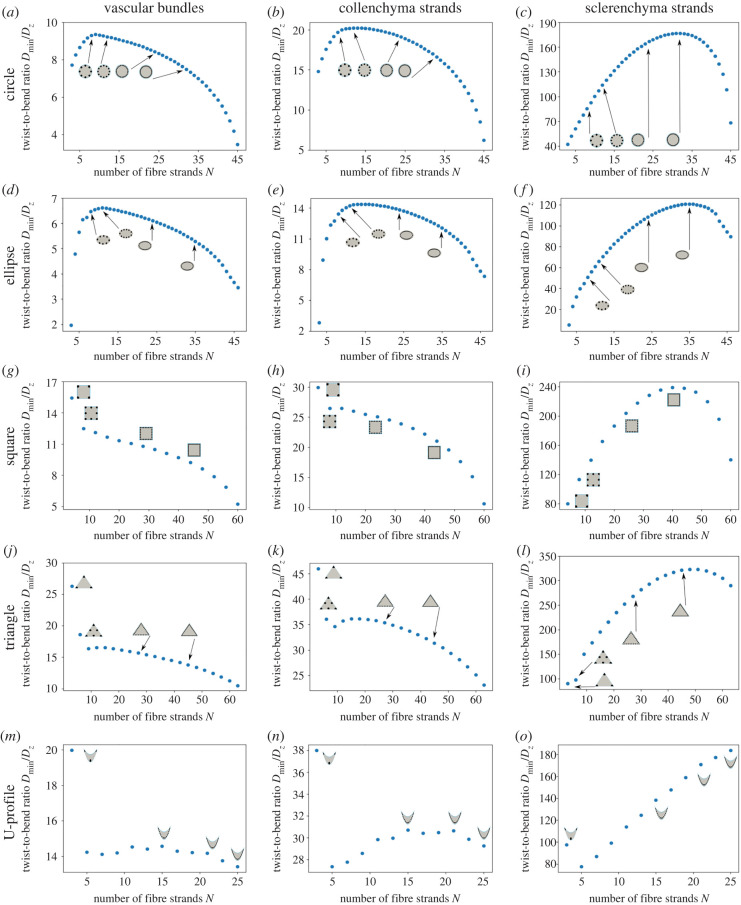
Evaluation of the twist-to-bend ratio for an increasing number of bundles/strands for various cross-sectional geometries (circle, ellipse, square, triangle, U-profile) and materials (vascular bundles, collenchyma strands, sclerenchyma strands). The circular (*a*–*c*), elliptic (*d*–*f*), triangular (*j*–*l*) and U-profiled (*m*–*o*) cross-sections start with three bundles/strands, whereas the square cross-sections (*g*–*i*) start with four bundles/strands.

Figure 7 shows the results of the mathematical calculations of the twist-to-bend ratio *D*_min_/*D*_*z*_ as a function of the number of individual fibres in circular, square, triangular, elliptic and U-profiled cross-sections. We increased the number of fibres according to the procedure described in §2.2, in which the total area of the fibre strands is fixed during the whole procedure. Thus, the number of fibre strands is the only free variable in this model and the twist-to-bend ratio is determined by a function *f*(*N*) depending only on the strand number *N*. The graphs of *D*_min_/*D*_*z*_ illustrate that an optimal number of fibre strands exists in order to achieve high twist-to-bend ratios that depend noticeably on the reference geometry and the material of the fibres. For cross-sections with corners (i.e. triangle, square, U-profile) and vascular bundles or collenchyma strands, the optimum is always reached for bundles/strands placed in the corners of the reference geometry. This is different for sclerenchyma fibres. Here *D*_min_/*D*_*z*_ first increases, with approximately linear growth, reaching a maximum for triangular and square cross-sections. For the U-profiled cross-section, this optimum is not reached, as more than 25 single bundles/strands would merge, and thus the procedure was stopped.

For circular and elliptic cross-sections, the behaviour is similar except that here the optimal number of bundles/strands for the achievement of high twist-to-bend ratios for vascular bundles and collenchyma strands is not determined by the number of corners. Furthermore, the charts of the twist-to-bend ratios are similar for elliptic and circular geometries, as the optimum number of bundles/strands varies only slightly for the three different materials. Nevertheless, the ratios at the optimum differ markedly; this can be explained because the twist-to-bend ratio *D*_min_/*D*_*z*_ includes the minimal flexural rigidity *D*_min_ along the principal axes. Because of symmetry, *D*_min_ is smaller for an elliptic geometry than for a circular geometry of the cross-section.

As the flexural rigidity remains nearly constant from a small number of bundles/strands, the chart of the twist-to-bend ratio is driven by the torsional rigidity, which first decreases almost linearly in *N* and, after reaching a minimum (for all cross-sections except the U-profiled), increases again (electronic supplementary material, S5 and figure S5.1). This behaviour is caused by two competing effects: a falling amplitude of Prandtl’s stress function *ϕ* around bundles/strands and a sieve effect caused by decreasing distances between single bundles/strands (see [9] for more details). Thereby, the number of bundles/strands for which the minimum torsional rigidity is reached depends decisively on the shape of the reference cross-section with *N* being the lowest for circular cross-sections and the highest for triangular cross-sections. Furthermore, the minimum of the torsional rigidity is approximately the same for all reference cross-sections. This is different for flexural rigidity, which differs noticeably between the reference geometries, thus resulting in a marked difference in the twist-to-bend ratio for circular, elliptic, U-profiled, square and triangular cross-sections; see figure 7 and electronic supplementary material, S5 and figure S5.1.

Comparing the values taken by *D*_min_/*D*_*z*_ for the optimal number of bundles/strands among all three materials, a trend emerges showing an increasing twist-to-bend ratio along the reference geometries in the order: elliptic, circular, square, U-profiled, triangular.

## 4. Discussion

### 4.1. Clusters in the rigidity charts

Since plants are simultaneously exposed to a wide range of bending and torsional loads, various morphological, anatomical and mechanical adaptations by means of the optimization of partly contradictory mechanical properties have developed during evolution [30]. Our previous investigations on the morphology and anatomy of plant stems have revealed a variety of cross-sectional geometries combined with various tissue patterns (figure 2). Furthermore, the geometric, mechanical and structural properties of entire axes could be determined in the framework of biomechanical tests (electronic supplementary material, S1 and table S1.1). The question remains as to what extent the morphological–anatomical features and/or mechanical properties are responsible for the structural properties of the plant stem. This study provides answers, because we have systematically analysed, by normalized artificial cross-sections, the simultaneous influence of the two-dimensional geometry of the entire plant stem and the cross-sectional arrangement of vascular bundles or strands of collenchyma and sclerenchyma on the flexural rigidity, the torsional rigidity and, thus, the trade-off between them, namely the dimensionless twist-to-bend ratio. High values of twist-to-bend ratio indicate that the plant axis has high flexural rigidity combined with low torsional rigidity. High flexural rigidity ensures that the plant axes remain upright, even under additional loads such as top loads of flowers and fruits, perching birds, passing animals, snow and rain. High torsional flexibility guarantees streamlining under wind load by twisting of the plant axis downwind with the supported of a gentle bending of the axis [2,7,16,19,20,31].

We placed our artificially designed cross-sections (figure 3) in rigidity charts defined by flexural and torsional rigidity. On the basis of the respective place in the rigidity chart, we provided information about the values of minimal flexural rigidity (along the *y*-axis), torsional rigidity (along the *x*-axis) and the twist-to-bend ratio (along the dashed diagonals representing constant twist-to-bend ratios) of the cross-sectional designs. We then defined clusters based on the arrangement of the individual designs. In 2003, Etnier also provided a stiffness mechanospace defined by flexural stiffness and torsional stiffness. She placed ideal beams characterized by their cross-sectional geometries and Poisson’s ratios into the mechanospace diagram and defined boundaries for possible extremes of botanical and zoological rod-shaped structures. However, the calculations did not take into account the cross-sectional tissue patterns [17].

For vascular bundles (figure 4), strands of collenchyma fibres (figure 5) and strands of sclerenchyma fibres (figure 6), we defined at least three clusters with respect to the position of the artificial designs in the rigidity charts: (1) cross-sections with a closed ring of vascular tissues, collenchyma or sclerenchyma in the periphery (*x*.3) exhibiting low twist-to-bend ratios (0.86−2.18); (2) cross-sections with a central position (*x*.1) also having low twist-to-bend ratios (0.85−2.99); (3) cross-sections with scattered bundles/strands in the parenchyma or individual bundles/strands in the periphery. In each of these three clusters, the angular cross-sections (square, triangle and U-profile) exhibited the highest twist-to-bend ratios. In addition to the three clusters found for all types of strengthening tissue, the square (*c*.7) and triangular designs (*d*.7), which had the sclerenchyma positioned in the corners, formed a fourth cluster with high twist-to-bend ratios of 80.07 and 90.02, respectively. All values are provided in table 2.

Because of the high degree of abstraction of our artificial cross-sections, engineers can also use the rigidity landscapes for the transfer of the cross-sectional geometry, together with the two-dimensional pattern of reinforcements, in technical applications (biomimetics). Moreover, the results of our simulation of artificial cross-sections provide a deeper understanding of the body plans of plants in the framework of reverse biomimetics [32].

In the following, we discuss the results of the mathematical simulations with respect to plant axes in general, and to the plant models shown in figure 2 in particular. Since the clusters are characterized by the tissue patterns and not by the cross-sectional geometry, we will focus on plant axes with similar tissue patterns. For the different geometries, we give a short overview of the plant families in which they mainly occur. With respect to the cross-sectional arrangement of the vascular tissue in stems and roots of higher plants, botanists distinguish between various types of stelar systems (= primary conductive tissue). In petioles, the vascular bundle distribution is often somewhat different from that in the stem. Wherever possible, we will refer to the various steles without going into detail, as our focus in this study is the mechanical aspects.

### 4.2. Cross-sectional geometries of plant axes

Most (upright) plant axes have an almost circular cross-sectional geometry (design *a*). The Opuntioideae, for example, a subfamily of the cactus family (Cactaceae), possess elliptic branches, so-called cladodes (design *b*). Square cross-sections (design *c*) are widespread in the mint family (Lamiaceae). Triangular cross-sections (design *d*) can be found in the grass families Poaceae, Cyperaceae and Juncaceae. U-profiled cross-sections (design *e*) are well known from petioles of Musaceae (e.g. banana, *Musa* sp.), Cannaceae (e.g. canna lily, *Canna* sp.), Strelitziaceae (e.g. bird-of-paradise, *Strelitzia reginae*) and plantain lilies (*Hosta* sp.).

### 4.3. Cross-sections with a central position of vascular bundles and fibre strands

*Design *x*.1*: Plant axes with a central position of the vascular tissues (= haplostele) or strengthening tissues (*x*.1) show low twist-to-bend ratios, which are not noticeably increased when replacing the vascular bundles by collenchyma and sclerenchyma strands. Change factors range between 0.91 and 1.01 (table 2). In rare cases, such as *b*.1 and *b*.3, the twist-to-bend ratios are even below 1.0 and the axes are easier to bend along the minor axis than to twist. The central position of the vascular bundles and fibre strands in plant axes is often interpreted as an adaptation to predominantly occurring tensile stresses, as in roots, climbing tendrils or plant organs occurring in flowing water. Because of their low flexural rigidity, these organs can bear only low bending stresses.

Dicotyledonous tap roots possess a central vascular cylinder surrounded by parenchyma and epidermis (*a*.1). They withstand shear and tensile stresses and are thus resistant to being pulled out of the soil. Roots thus firmly anchor the plant in the soil [33]. Tendrils (*a*.1) of the climbing plants *Parthenocissus tricuspidata* [34] and *Passiflora discophora* [35] and attachment roots (*d*.1) of the climbing *Vanilla* spp. and English ivy [36], which are predominantly under tensile loads, also have a core of vascular bundles.

In addition, some aquatic plants, which are subjected to tensile stresses from water flow, show a central positioning of the vascular tissues embedded in aerenchyma and parenchyma and surrounded by epidermis. The earliest land plants in the late Silurian/early Devonian, such as *Cooksonia* sp., *Rhynia gwynne-vaughanii*, *Aglaophyton major* and *Zosterophyllum* sp., had aerial stems with a central core of vascular tissue, which might be reminiscent of their ancestors that lived in water [37,38].

### 4.4. Cross-sections with a closed peripheral ring of vascular bundles and fibre strands

*Design x.3*: A closed ring of vascular tissues (= siphonostele) or strengthening tissue increases the torsional rigidity and contributes even more to high flexural rigidity the further the ring is positioned to the periphery.

An intriguing example is the growth form of lianas, such as *Aristolochia macrophylla*. Young lianas build circular searcher stems that have circular cross-sections and a peripheral closed ring of sclerenchyma, and that are therefore (very) rigid in bending and torsion (*a*.3). These searcher stems are mechanically self-supporting. Once attachment is secured, secondary growth processes are intensified and, during growth, the secondary xylem and phloem increasingly take up space within the ring, causing radial and tangential stresses and thus leading to cracks in the ring. The cracks are sealed with parenchyma cells, leading to fragmentation of the ring. Thus, older liana stems that are secured to the host tree possess, in addition to the very flexible core of ‘lianescent’ wood, a fragmented ring of sclerenchyma (*a*.6), which is however much more flexible in bending and torsion [36,39].

Comparisons of free-standing and tree-supported climbing stems of *Croton pullei* have shown that the larger the diameter, the greater the differences in the twist-to-bend ratio. Free-standing stems possess dense wood with small-lumen vessels, whereas tree-supported stems have a much higher proportion of large diameter vessels in their ‘lianescent’ wood. In the highest class of stem diameter (15.1–20 mm), young free-standing stems have twist-to-bend ratios of 10.3 and older non-self-supporting stems have ratios of 6.5 [40].

Soffiati & Rowe [41] have investigated the climbing cactus *Selenicereus setaceus*, the stems of which have in common a core of vascular tissue (*x*.1), a stiff dermal tissue represented by a hypodermis and, in older stems, also a peridermal coverage (*x*.3), but various cross-sectional geometries at different stages of growth. The cactus has cylindrical to elliptic basal stems with a cylinder of secondary wood, triangular climbing stems with a core of vascular tissue and apical star-shaped stems with centrally positioned vascular tissue that search for new supports. The three different types of stem differ significantly with respect to the axial second moment of area and the elastic modulus. The flexural rigidity of the basal circular and the apical star-like stem parts do not differ significantly. However, the flexural rigidity of the triangular stems is significantly smaller, i.e. the basal (supporting) stem parts and apical searching stems are stiffer in bending than are the climbing stem parts [41].

Another example is the elliptic to U-profiled cross-sections of *Hemigraphis alternata* petioles (figure 2*e*,*f*) that combine a U-profiled arrangement of five vascular strands along the major axis with a closed ring of collenchymatous hypodermis in the periphery (*b*.3). The petioles exhibit a twist-to-bend ratio of approximately 12; i.e. they are 12 times more rigid in bending than in torsion. These petioles bear a lamina that weighs, at median, 7.5 times as much as the leaf stalk itself [1] (electronic supplementary material, S1 and table S1.1).

Square cross-sections of flower stalks of *Leonurus cardiaca* (figure 2*g*,*h*) combine a peripheral ring of strengthening tissue (*c*.3) with a corner position of collenchyma (*d*.7). Kaminski *et al.* [24] showed that, during ontogeny, the area sum of collenchyma fibres is characterized by clear negative allometric scaling (*α* = 0.721). By contrast, the area sums of the vascular tissue (*α* = 1.098) and parenchyma (*α* = 1.071) reveal moderate positive allometric growth. In other words, vascular tissue and parenchyma grow at a faster rate than the other tissues of the internode. The ontogenetic changes are also reflected when comparing internodes from different heights above ground and in samples from June and September. Spatial comparisons of the second and third apical internodes and temporal comparisons of the respective internodes in June and September have revealed median twist-to-bend ratios between ≈14 and ≈19, with none of the four possible comparisons showing a significant difference. By contrast, the absolute values of flexural rigidity and torsional rigidity increase significantly from the second and third apical internodes measured in the same months or for the same internode measured in June and September, respectively (electronic supplementary material, S1 and table S1.1).

### 4.5. Cross-sections with individual vascular bundles and fibre strands

The tissue patterns of cross-sections with individual strands of vascular and strengthening tissue form another cluster, which includes designs *x*.2, *x*.4–*x*.6 and partly *x*.7.

*Design x.2*: The star-shaped arrangement of vascular tissue in the cross-section, the so-called actinostele (ancient Greek ακτινοτός, which means surrounded by rays), can be found in the aerial stems of the Lycopodiopsida and in the central cylinder of the roots of some cormophytes (*a*.2). Compared with design *x*.1 the star-shaped core of design *x*.2 increases the flexural rigidity (electronic supplementary material, S3 and table S3.1) as a result of the displacement of the strengthening material into the periphery. Designs *x*.1 and *x*.2 exhibit comparable values of torsional rigidity with respect to vascular tissues (figure 4) but lower values with respect to collenchyma (figure 5) and sclerenchyma (figure 6). A small change in the tissue pattern from circular (*x*.1) to star-shaped (*x*.2) leads, with the same material input, to a substantial increase in the twist-to-bend ratio (table 2). This increase cannot be found in land plants with a star-shaped actinostele, for example in *Asteroxylon mackiei*. In these early lycopsids, the indentations of the actinostele are relatively shallow, so that no significant increase in flexural rigidity occurs compared with circular cross-sections. The ‘selective advantage’ of actinosteles compared with haplosteles in early land plants is their higher surface-to-volume ratio, i.e. the larger contact area with the surrounding parenchyma. This facilitates and improves the exchange of water between the water-conducting actinostele and the parenchyma, which serves as the main stabilizing tissue in these turgor-stabilized plants [37,38].

*Design x.4*: The succulent leaves of the genus *Delosperma* show an arrangement of centrally positioned vascular bundles combined with several peripheral individual bundles. The leaves of *Delosperma cooperi* are circular (*a*.4) to oval (*b*.4), whereas the leaves of *Delosperma ecklonis* have a triangular geometry (*d*.4) [13,42]. With respect to table 2 the twist-to-bend ratios of *a*.4 and *b*.4 are approximately half the value of that of *d*.4. These differences are the result of the smaller flexural rigidity and higher torsional rigidity of *a*.4 and *b*.4 compared with *d*.4. From a technical point of view, *Delosperma* leaves can be described as a five-shell model, the layers of which have various thicknesses and are alternately under pre-tension (epidermis, ring of individual strands of vascular tissue, central strand of vascular bundles) and pre-compression (outer chlorenchyma, inner hydrenchyma). These pre-stresses play a major role in the sealing of damage when the entire leaf bends or contracts until the wound edges meet [43,44]. Since only the ratios of the layer thicknesses and elastic moduli of the five tissues are relevant for the sealing mechanism, a transfer into technical materials is possible [43].

*Design x.5*: Monocotyledonous stems mostly show randomly scattered vascular bundles in the parenchyma, forming the so-called atactostele (ancient Greek άτακτος, which means disordered). Although *Pilea peperomioides* is a dicotyledonous plant, the cross-sectional tissue pattern of its petioles can be compared most closely to that of artificial design *a*.5. However, the vascular tissues are not randomly scattered in the parenchyma, but lie more in the centre of the circular cross-section (figure 2*a*,*b*). Petioles of *P. peperomioides* exhibit twist-to-bend ratios of ≈13 [1] (electronic supplementary material, S1 and table S1.1). Mechanical tests of the transition zone between the petiole and the lamina, however, reveal median twist-to-bend ratios of 0.67, which means that the transition zones are easier to bend than to twist [5].

According to table 2 the complete replacement of the vascular tissue by collenchyma results in a doubling of the twist-to-bend ratio (change factor: 2.01–2.14) for all investigated geometries. If the vascular tissue is completely replaced by sclerenchyma, the change factor lies between 7.85 for U-profiles and 12.36 for circles.

*Design x.6*: With respect to the mathematical calculations shown in table 2, the twist-to-bend ratio of design *x*.6 with individual peripheral reinforcement strands is approximately doubled if all vascular bundles are replaced by collenchyma (change factor: 2.06–2.23). If the vascular bundles are completely replaced by sclerenchyma fibres, the change factor is between 9.24 (U-profile) and 14.43 (circle). Further simulations show that the twist-to-bend ratio depends on the number of individual strands in the periphery (figure 7). With the exception of small numbers of peripheral strands, the flexural rigidity is almost constant, whereas the torsional rigidity decreases with an increasing number of strands and the total volume ratio of bundles being kept constant (electronic supplementary material, S5 and figure S5.1). In other words, the twist-to-bend ratio is determined by the torsional rigidity, which decreases with increasing numbers of peripheral reinforcement strands.

Design *e*.6 is represented by the U-profiled petioles of *H. tardiana* (figure 2*k*,*l*) equipped with a median of 23 vascular bundles. The U-profile is special in the sense that the median ratio *I*/*K* = 1.08, which means that the geometric properties increase the twist-to-bend ratio slightly [1]. With the exception of the U-profile with three bundles, which show extremely high twist-to-bend ratios, the values are much smaller and slightly decrease with increasing bundles (figure 7*m*) (electronic supplementary material, S1 and table S1.1).

However, in real plants, not all vascular bundles are replaced by strengthening tissue. Instead, plant axes with scattered vascular bundles (*x*.5) in the parenchyma are additionally strengthened by peripheral reinforcement strands (*x*.6). Investigations of *C. bicolor* petioles (figure 2*c*,*d*) have revealed that their mechanical properties exhibit a high value of *Ē*/*$\bar{G}$* ≈ 64, resulting in a twist-to-bend ratio of approximately 40 [1] (electronic supplementary material, S1 and table S1.1). The almost circular petioles have in the periphery a median of 66 individual collenchyma strands, which are elliptical in cross-section [23]. By contrast, figure 7*b* shows that, according to our calculations, 45 collenchyma strands already merge. This difference between 44 and 66 individual strands is caused by the geometry of the strands, which are elliptical in the petiole but are assumed to be circular in our calculation, and by the different percentage area of the collenchyma strands with A=4.75% in the petiole and A=15% in the artificial cross-section.

Investigations of the internodes of the flower stalk of *C. pendula* (figure 2*i*,*j*) have revealed peak values of the twist-to-bend ratio ever measured for herbaceous plant axes. With a median of *I*/*K* = 0.99, the geometric properties only minimally reduce the twist-to-bend ratio. The huge median values of *Ē*/$\bar{G}$ ≈ 179 result in extremely high twist-to-bend ratios ranging between 85 and 403 [21]. Based on these results, Wolff-Vorbeck *et al.* [9] carried out simulations on triangular cross-sections consisting of vascular bundles scattered in the parenchyma and numerous strands of collenchyma or sclerenchyma in the periphery with surrounding epidermis. Similar to the results in this study, and depending on the number of sclerenchyma strands, the flexural rigidity is almost constant and the torsional rigidity shows a U-shaped curve (see [9, figs 4 and 5]). The twist-to-bend ratio is maximal when 49 peripheral sclerenchyma strands are added to the triangular cross-section. This optimum of 49 strands found in the simulation corresponds well with the average value of 49 ± 8, which is calculated from the numbers of strands of the apical and more basal internodes of real *C. pendula* plants [21].

### 4.6. Cross-sections with a corner position of fibre strands

*Design x.7*: Square cross-sections with four strands (*c*.7) and triangular cross-sections with three strands (*d*.7) of collenchyma or vascular bundles at the corners exhibit the highest twist-to-bend ratios, which decrease with an increasing number of bundles/strands (figure 7*d*,*e*,*g*,*h*). On the contrary, squares and triangles with sclerenchyma strands in the corners have the lowest twist-to-bend ratio compared with designs *c*.6 and *d*.6, which possess numerous strands and form their own clusters (figure 6). Since *L. cardiaca* is a combination of designs *c*.6 and *c*.7, we discussed it in the above section.

## 5. Conclusion

The ultimate aim of our study has been to find answers to the scientific question: How do the cross-sectional geometry and/or the arrangement of vascular bundles or strengthening tissues influence the flexural rigidity, the torsional rigidity and thus the twist-to-bend ratio of plant axes? As answers, we can derive from our research the following general statements, which give an in-depth understanding of the functional morphology of plants and might provide a basis for engineers developing bioinspired designs.

1. On the basis of our mathematical calculations and experimental research, we can summarize our results as follows.
  1.1. Flexural rigidity (*ĒI*) and torsional rigidity (*ḠK*) are determined by geometric characteristics (axial second moment of area *I* and torsion constant *K*) and effective mechanical properties (elastic modulus *Ē* and torsional modulus *Ḡ*).
  1.2. Flexural rigidity increases when the vascular bundles or strengthening tissues are placed more peripherally.
  1.3. Torsional rigidity decreases the more the vascular bundles or strengthening tissues are separated.
  1.4. The twist-to-bend ratio (*ĒI*/*ḠK*) is a trade-off between flexural and torsional rigidity.
  1.5. Plant axes with high twist-to-bend ratios are stiff enough to remain upright under bending loads and are flexible enough to twist under torsion loads.
  1.6. Plant axes with a twist-to-bend ratio *ĒI*/*ḠK* of less than 1.0 are particularly stiff in torsion, since their torsional rigidity is greater than their flexural rigidity.
  1.7. High ratios of effective elastic modulus and effective torsional modulus (*Ē*/*Ḡ*) result in high twist-to-bend ratios.
  1.8. Cross-sectional geometry can contribute to high twist-to-bend ratios if the ratio of the axial second moment of area and torsion constant *I*/*K* is greater than 1.0.
2. Conclusions from the rigidity charts of all artificial cross-sections in which the flexural rigidity is shown as a function of the torsional rigidity can be summarized as follows.
  2.1. Artificial cross-sections with the same basic cross-sectional geometry do not cluster in the rigidity chart.
  2.2. Clusters are found for artificial cross-sections with a closed ring of vascular bundles or strengthening tissues in the periphery exhibiting low twist-to-bend ratios.
  2.3. Clusters are found for artificial cross-sections with a central position of vascular bundles or strengthening tissues with low twist-to-bend ratios.
  2.4. Clusters are found for artificial cross-sections with individual strands of strengthening tissue or vascular bundles.
  2.5. A fourth cluster is found for square and triangular cross-sections with sclerenchyma in the stem corners.
3. The number of individual bundles/strands influences the structural properties as follows.
  3.1. With the exception of small numbers of peripheral bundles/strands, the flexural rigidity is almost constant with an increasing number of vascular bundles or strands of strengthening tissues.
  3.2. With the exception of small numbers of peripheral bundles/strands, the torsional rigidity initially decreases almost linearly and, after reaching a minimum, increases again with an increasing number of bundles/strands. This behaviour is caused by two competing effects: a falling amplitude of Prandtl’s stress function *ϕ* around bundles/strands and the sieve effect caused by decreasing distances between single bundles/strands.
  3.3. The minimum values of the torsional rigidity are almost the same for all geometries, whereas the values of the flexural rigidity differ between the geometries.
  3.4. The twist-to-bend ratio is determined, on the one hand, by the torsional rigidity, which decreases with increasing numbers of bundles/strands to similar minimum values for all geometries and, on the other hand, by the different values of flexural rigidity, which in turn depend on the geometry.
  3.5. The maximum and minimum values of the twist-to-bend ratio are achieved with different numbers of individual bundles/strands, depending on the respective cross-sectional geometry and on whether vascular bundles, collenchyma strands or sclerenchyma strands are considered.

## Data Availability

The data that support the findings of this study are openly available in the electronic supplementary material [45].

## References

[1] Langer M, Kelbel M, Müller C, Speck T, Speck O. 2021 Twist-to-bend ratios and safety factors of petioles having various geometries, shapes and sizes. Front. Plant Sci. **12**, 2586. (10.3389/fpls.2021.765605) PMC8632552 34858462

[2] Vogel S. 1992 Twist-to-bend ratios and cross-sectional shapes of petioles and stems. J. Exp. Bot. **43**, 1527-1532. (10.1093/jxb/43.11.1527)

[3] Drost HG, Janitza P, Grosse I, Quint M. 2017 Cross-kingdom comparison of the developmental hourglass. Curr. Opin. Genet. Dev. **45**, 69-75. (10.1016/j.gde.2017.03.003) 28347942

[4] Rowe NP, Speck T. 1998 Biomechanics of plant growth forms: the trouble with fossil plants. Rev. Palaeobot. Palynol. **102**, 43-62. (10.1016/S0034-6667(98)00013-X)

[5] Langer M, Hegge E, Speck T, Speck O. 2021 Acclimations to wind loads and/or contact stimuli? A biomechanical study of peltate leaves of *Pilea peperomioides*. J. Exp. Bot. **73**, Erab541. 10.1093/jxb/erab541 PMC8866637 34893822

[6] Young WC, Budynas RG, Sadegh AM. 2002 Roark’s formulas for stress and strain, vol. 7. New York, NY: McGraw-Hill.

[7] Niklas KJ. 1992 Plant biomechanics: an engineering approach to plant form and function. Chicago, IL: University of Chicago Press.

[8] Wolff-Vorbeck S, Langer M, Speck O, Speck T, Dondl P. 2019 Twist-to-bend ratio: an important selective factor for many rod-shaped biological structures. Sci. Rep. **9**, 17182. (10.1038/s41598-019-52878-z) 31748548 PMC6868162

[9] Wolff-Vorbeck S, Speck O, Speck T, Dondl P. 2021 Influence of structural reinforcements on the twist-to-bend ratio of plant stems. Sci. Rep. **11**, 212323. (10.1038/s41598-021-00569-z) PMC8551206 34707194

[10] Speck T, Schmitt M. 1992 Mechanische Werte. In *Lexikon der Biologie – Biologie im Überblick* (ed. M Schmitt), pp. 244–247. Freiburg, Germany: Herder.

[11] Bold G, Langer M, Börnert L, Speck T. 2020 The protective role of bark and bark fibers of the giant sequoia (*Sequoiadendron giganteum*) during high-energy impacts. Int. J. Mol. Sci. **21**, 3355. (10.3390/ijms21093355) PMC7247593 32397436

[12] Ambronn H. 1881 Über die Entwickelungsgeschichte und die mechanischen Eigenschaften des Collenchyms: Ein Beitrag zur Kenntniss des mechanischen Gewebesystems. Jahrbücher für wissenschaftliche Botanik. In *Jahrbücher für wissenschaftliche Botanik*, vol. 12, pp. 473–541. G. Bernstein.

[13] Speck O, Schlechtendahl M, Borm F, Kampowski T, Speck T. 2018 Humidity-dependent wound sealing in succulent leaves of *Delosperma cooperi*—an adaptation to seasonal drought stress. Beilstein J. Nanotechnol. **9**, 175-186. (10.3762/bjnano.9.20) 29441263 PMC5789399

[14] Mylo MD, Hesse L, Masselter T, Leupold J, Drozella K, Speck T, Speck O. 2021 The morphology and anatomy of branch-branch junctions in Opuntioideae: a comparative study supported by mechanical tissue quantification. Plants **10**, 2313. (10.3390/plants10112313) 34834679 PMC8618873

[15] Bargel H, Spatz HC, Speck T, Neinhuis C. 2004 Two-dimensional tension tests in plant biomechanics—sweet cherry fruit skin as a model system. Plant Biol. **6**, 432-439. (10.1055/s-2004-821002) 15248126

[16] Ennos AR. 1993 The mechanics of the flower stem of the sedge *Carex acutiformis*. Ann. Bot. **72**, 123-127. (10.1006/anbo.1993.1089)

[17] Etnier SA. 2003 Twisting and bending of biological beams: distribution of biological beams in a stiffness mechanospace. Biol. Bull. **205**, 36-46. (10.2307/1543443) 12917220

[18] Vogel S. 2007 Living in a physical world XI. To twist or bend when stressed. J. Biosci. **32**, 643-655. (10.1007/s12038-007-0064-6) 17762137

[19] Ennos AR, Spatz H, Speck T. 2000 The functional morphology of the petioles of the banana, *Musa textilis*. J. Exp. Bot. **51**, 2085-2093. (10.1093/jexbot/51.353.2085) 11141182

[20] Ahlquist S, Kampowski T, Torghabehi OO, Menges A, Speck T. 2015 Development of a digital framework for the computation of complex material and morphological behavior of biological and technological systems. Comput.-Aid. Des. **60**, 84-104. (10.1016/j.cad.2014.01.013)

[21] Speck O, Steinhart F, Speck T. 2020 Peak values of twist-to-bend ratio in triangular flower stalks of *Carex pendula*: a study on biomechanics and functional morphology. Am. J. Bot. **107**, 1-9. (10.1002/ajb2.1558) 33190221

[22] Niklas KJ. 1999 A mechanical perspective on foliage leaf form and function. New Phytol. **143**, 19-31. (10.1046/j.1469-8137.1999.00441.x)

[23] Caliaro M, Schmich F, Speck T, Speck O. 2013 Effect of drought stress on bending stiffness in petioles of *Caladium bicolor* (Araceae). Am. J. Bot. **100**, 2141-2148. (10.3732/ajb.1300158) 24190949

[24] Kaminski R, Speck T, Speck O. 2017 Adaptive spatiotemporal changes in morphology, anatomy, and mechanics during the ontogeny of subshrubs with square-shaped stems. Am. J. Bot. **104**, 1157-1167. (10.3732/ajb.1700110) 28814404

[25] Langer M, Speck T, Speck O. 2021 The transition zone between petiole and lamina: a functionally crucial but often overlooked leaf trait. Plants **10**, 774. (10.3390/plants10040774) 33920846 PMC8071152

[26] Mora MG, Müller S. 2003 Derivation of the nonlinear bending-torsion theory for inextensible rods by Γ-convergence. Cal. Var. Partial Differ. Equ. **18**, 287-305. (10.1007/s00526-003-0204-2)

[27] Ecsedi I. 2005 Bounds for the effective shear modulus. Eng. Trans. **53**, 415-423.

[28] Neukamm S. 2012 Rigorous derivation of a homogenized bending-torsion theory for inextensible rods from three-dimensional elasticity. Arch. Rational Mech. Anal. **206**, 645-706. (10.1007/s00205-012-0539-y)

[29] Hejnowicz Z, Sievers A. 1995 Tissue stresses in organs of herbaceous plants: II. Determination in three dimensions in the hypocotyl of sunflower. J. Exp. Bot. **46**, 1045-1053. (10.1093/jxb/46.8.1045)

[30] Lambers H, Chapin III FS, Pons TL. 2008 Plant physiological ecology, pp. 4-6. New York, NY: Springer Science & Business Media.

[31] Etnier SA, Vogel S. 2000 Reorientation of daffodil (Narcissus: amaryllidaceae) flowers in wind: drag reduction and torsional flexibility. Am. J. Bot. **87**, 29-32. (10.2307/2656682) 10636827

[32] International Organization for Standardization. 2015 *Biomimetics–terminology, concepts and methodology*. Berlin,Germany: Beuth.

[33] Ennos R. 2011 Solid biomechanics. Princeton, NJ: Princeton University Press.

[34] Steinbrecher T, Beuchle G, Melzer B, Speck T, Kraft O, Schwaiger R. 2011 Structural development and morphology of the attachment system of *Parthenocissus tricuspidata*. Int. J. Plant Sci. **172**, 1120-1129. (10.1086/662129)

[35] Klimm F, Schmier S, Bohn HF, Kleiser S, Thielen M, Speck T. 2022 Biomechanics of tendrils and adhesive pads of the climbing passion flower *Passiflora discophora*. J. Exp. Bot. **73**, 1190-1203. (10.1093/jxb/erab456) 34673926 PMC8866636

[36] Rowe NP, Speck T. 2015 Stem biomechanics, strength of attachment, and developmental plasticity of vines and lianas. In Ecology of lianas (eds SA Schnitzer, F Bongers, RJ Burnham, FE Putz), pp. 323-341. Chichester, UK: Wiley.

[37] Speck T, Vogellehner D. 1988 Biophysical examinations of the bending stability of various stele types and the upright axes of early ‘vascular’ land plants. Bot. Acta **101**, 262-268. (10.1111/j.1438-8677.1988.tb00042.x)

[38] Bateman RM, Crane PR, DiMichele WA, Kenrick PR, Rowe NP, Speck T, Stein WE. 1998 Early evolution of land plants: phylogeny, physiology, and ecology of the primary terrestrial radiation. Annu. Rev. Ecol. Syst. **29**, 263-292. (10.1146/annurev.ecolsys.29.1.263)

[39] Busch S, Seidel R, Speck O, Speck T. 2010 Morphological aspects of self-repair of lesions caused by internal growth stresses in stems of *Aristolochia macrophylla* and *Aristolochia ringens*. Proc. R. Soc. B **277**, 2113-2120. (10.1098/rspb.2010.0075) PMC2880149 20236971

[40] Gallenmüller F, Müller U, Rowe N, Speck T. 2001 The growth form of *Croton pullei* (Euphorbiaceae) – functional morphology and biomechanics of a neotropical liana. Plant Biol. **3**, 50-61. (10.1055/s-2001-11750)

[41] Soffiatti P, Rowe NP. 2020 Mechanical innovations of a climbing cactus: functional insights for a new generation of growing robots. Front. Rob. AI **7**, 64. (10.3389/frobt.2020.00064) PMC7806016 33501232

[42] Hesse L, Kampowski T, Leupold J, Caliaro S, Speck T, Speck O. 2020 Comparative analyses of the self-sealing mechanisms in leaves of *Delosperma cooperi* and *Delosperma ecklonis* (Aizoaceae). Int. J. Mol. Sci. **21**, 5768. (10.3390/ijms21165768) PMC7460835 32796721

[43] Konrad W, Flues F, Schmich F, Speck T, Speck O. 2013 An analytic model of the self-sealing mechanism of the succulent plant *Delosperma cooperi*. J. Theor. Biol. **336**, 96-109. (10.1016/j.jtbi.2013.07.013) 23907028

[44] Klein H, Hesse L, Boljen M, Kampowski T, Butschek I, Speck T, Speck O. 2018 Finite element modelling of complex movements during self-sealing of ring incisions in leaves of *Delosperma cooperi*. J. Theor. Biol. **458**, 184-206. (10.1016/j.jtbi.2018.08.023) 30149008

[45] Wolff-Vorbeck S, Speck O, Langer M, Speck T, Dondl PW. 2022 Charting the twist-to-bend ratio of plant axes. *Figshare*. (10.6084/m9.figshare.c.6032484) PMC9214286 35730171

